# A Feature-Driven Decision Support System for Heart Failure Prediction Based on *χ*^2^ Statistical Model and Gaussian Naive Bayes

**DOI:** 10.1155/2019/6314328

**Published:** 2019-11-20

**Authors:** Liaqat Ali, Shafqat Ullah Khan, Noorbakhsh Amiri Golilarz, Imrana Yakubu, Iqbal Qasim, Adeeb Noor, Redhwan Nour

**Affiliations:** ^1^School of Information and Communication Engineering, University of Electronic Science and Technology of China (UESTC), Chengdu 611731, China; ^2^Department of Electrical Engineering, University of Science and Technology, Bannu 28100, Pakistan; ^3^Department of Electronics, University of Buner, Buner 17290, Pakistan; ^4^School of Computer Science and Engineering, University of Electronic Science and Technology of China (UESTC), Chengdu 611731, China; ^5^Department of Computer Science, University of Science and Technology, Bannu 28100, Pakistan; ^6^Department of Information Technology, Faculty of Computing and Information Technology, King Abdulaziz University, Jeddah 80221, Saudi Arabia; ^7^Department of Computer Science, Taibah University, Medina 42353, Saudi Arabia

## Abstract

Heart failure (HF) is considered a deadliest disease worldwide. Therefore, different intelligent medical decision support systems have been widely proposed for detection of HF in literature. However, low rate of accuracies achieved on the HF data is a major problem in these decision support systems. To improve the prediction accuracy, we have developed a feature-driven decision support system consisting of two main stages. In the first stage, *χ*^2^ statistical model is used to rank the commonly used 13 HF features. Based on the *χ*^2^ test score, an optimal subset of features is searched using forward best-first search strategy. In the second stage, Gaussian Naive Bayes (GNB) classifier is used as a predictive model. The performance of the newly proposed method (*χ*^2^-GNB) is evaluated by using an online heart disease database of 297 subjects. Experimental results show that our proposed method could achieve a prediction accuracy of 93.33%. The developed method (i.e., *χ*^2^-GNB) improves the HF prediction performance of GNB model by 3.33%. Moreover, the newly proposed method also shows better performance than the available methods in literature that achieved accuracies in the range of 57.85–92.22%.

## 1. Introduction

Heart failure (HF) is a condition whereby the heart is unable to supply enough blood to satisfy the body's requirements. The coronary artery as an integral part of the heart is accountable for supplying blood to the heart. Coronary artery disease (narrowed or blocked arteries) is the most prevalent type of heart disease and the most common cause of HF [1].

There are many imperilling conditions that result in a HF disease. These conditions can be put into two categories, with the first category consisting of risk or imperilling conditions that cannot be altered, e.g., patient's sex, age, and family history. The second category, which can be altered, consists of conditions that are attributed to the way of life of the patient, for instance, smoking habit, high cholesterol level, high level of blood pressure, and physical inactivity [2]. In addition, prevalent HF symptoms include dyspnea (shortage of breath), edema (swollen feet), fatigue, and weakness.

With so many factors to be analyzed, HF management becomes very complicated and even worse, particularly in nations that lack appropriate diagnostic instruments and medical experts [3, 4]. Furthermore, different tests are recommended by health practitioners to diagnose HF disease. Some of these tests are electrocardiogram (ECG), nuclear scan, angiography, and echocardiogram [5]. Among these tests, ECG is a noninvasive technique [6, 7]. But it is not very effective as it may lead to undiagnosed symptoms of HF disease [5]. This factor leads to angiography, a sort of diagnosis used to verify instances of heart disease. It is considered as the finest approach for HF disease diagnosis. However, some problems are associated with it such as its side effects, high cost, and requirement of high level of technical expertise [8, 9]. Thus, alternative modalities are needed which can solve these problems. It is therefore necessary to develop an efficient, intelligent, medical decision-making support system with the principles of data mining and machine learning.

In literature, various decision support systems with regards to support vector machine (SVM), decision tree, k-nearest neighbor (KNN), fuzzy logic based algorithms, artificial neural network (ANN), and ensembles of ANN have been suggested for the prediction of HF disease [1, 2, 10–17]. Robert Detrano, who gathered HF-related information for the Cleveland heart disease data, used logistical regression to predict HF risk assessment, achieving a classification precision of 77%. Newton Cheung verified the feasibility of various classifiers including C4.5, Naive Bayes, BNND, and BNNF algorithms and achieved 81.11%, 81.48%, 81.11%, and 80.95%, respectively, as HF risk prediction accuracies. Polat et al. [18] used a decision support system that utilized artificial immune system (AIS) and produced 84.5% of classification accuracy. Özşen and Güneş [19] developed a modified AIS and obtained an HF risk prediction accuracy of 87.43%. A model of neural network ensemble was proposed by Das et al. [2] to enhance the classification precision and obtained a percentage of 89.01 for HF prediction accuracy. Samuel et al. [20] proposed an embedded decision support scheme on the basis of ANN and Fuzzy AHP and obtained a 91.10% HF risk classification accuracy. Ali et al. suggested a machine learning method by stacking and optimizing two SVMs together for improved HF risk prediction and obtained HF prediction performance of 92.22% [21]. Paul et al. have recently established an adaptive weighted fuzzy system ensemble-based model. Their suggested model led to a 92.31% HF prediction accuracy.

Inspired from the various decision support systems proposed earlier and discussed above, we also attempted to develop a new decision support system for HF risk prediction with an aim to improve classification accuracy and reduce computational cost or complexity. The decision support system developed in this study is named *χ*^2^-GNB. The *χ*^2^-GNB model uses *χ*^2^ statistical model to rank features according to *χ*^2^ test score. To obtain an ideal (i.e., optimal) number of the ranked features in this paper, we exploit the forward best-first search approach. The performance of each of the generated subset of features is evaluated using GNB model which is used as a machine learning classifier. It is worth discussing that the suggested *χ*^2^-GNB model utilizes a simple predictive model but exhibits better efficiency than more complex predictive models such as ANN and even ensembles of ANNs. No prior research, to the best of our awareness, addressed the hybridization of GNB model from the family of Naive Bayes classifiers with *χ*^2^ model for the detection of HF disease. Compared with other techniques in the literature, the experimental findings of the suggested method are promising in terms of HF risk prediction accuracy.

The remainder of the manuscript is constructed as follows: Section 2 describes the materials (dataset) and the suggested methods.  Section 3 gives a discussion of metrics used for evaluation and validation.  Section 4 presents the findings of experiments and discussion.  Section 5, as the final part, concludes the paper.

## 2. Materials and Methods

### 2.1. Dataset Description

The University of California, Irvine (UCI) provides an online repository for machine learning from which the Cleveland heart disease dataset was obtained for experiments in this paper. 297 samples out of 303 samples contained in the dataset include no missing values, whereas 6 cases possess missing features' values. The data, in its original form, have 76 features. However, all the published research studies only refer to 13 of them. These commonly used 13 features are tabulated in Table 1.

### 2.2. Proposed Method

The suggested method, i.e., *χ*^2^-GNB comprises two phases. The first phase ranks features with the use of the *χ*^2^ statistical model. Amidst each positive feature *f*_*i*_ and class, i.e., *θ*, *χ*^2^ statistics are calculated using the *χ*^2^ model. That is, the model performs *χ*^2^ test which measures dependence between each feature and class. This approach, therefore, identifies those features (attributes) that most probably are not class-dependent. Thus, these features (attributes) are regarded as irrelevant for classification. The process of features selection itself is done in two phases. Phase one deals with the raking of features based on the *χ*^2^ test score while the second phase considers the search for ideal subset of features (attributes) from the available ranked features. It is worth mentioning that the feature ranking process is done using training data only, i.e., testing data are kept aside in order to avoid bias. Prior to the ranking of features and selection, data partitioning is performed. The features are ranked and selected on the basis of training data. The same attributes are also selected for the testing data during the validation phase or testing process. The process of ranking the features follows the basis of *χ*^2^ test which is expressed as follows.

For a classification that is binary in nature and contains *τ* instances, a positive and a negative class (two classes), we can construct Table 2 to compute the *χ*^2^ test score.

(1) $$\begin{array}{c} E_{\alpha }=\left(\alpha +\beta \right)\frac{\alpha +\beta }{\tau }. \end{array}$$

Similarly, *E*_*β*_, *E*_*λ*_, and *E*_*γ*_ can also be computed. According to the general *χ*^2^ test formulation, we have

(2) $$\begin{array}{c} \chi^{2}=\frac{1}{d}\overset{n}{\underset{k=1}{\sum }}\frac{{\left(O_{k}-E_{k}\right)}^{2}}{E_{k}},\\ \chi^{2}=\frac{{\left(\alpha -E_{\alpha }\right)}^{2}}{E_{\alpha }}+\frac{{\left(\beta -E_{\beta }\right)}^{2}}{E_{\beta }}+\frac{{\left(\lambda -E_{\lambda }\right)}^{2}}{E_{\lambda }}+\frac{{\left(\gamma -E_{\gamma }\right)}^{2}}{E_{\gamma }}. \end{array}$$

Readers can refer to [22] more enlightenment on the use of *χ*^2^ statistics for selecting and discretizing features. After features ranking by the above *χ*^2^ test score, we need to search the optimized subset of features based on the *χ*^2^ test. This is done by exploiting the forward best-first search algorithm. That is, first of all, we select solitarily, a feature having the greatest *χ*^2^ test score and then use the GNB predictive model to check its performance. In next iteration, we add another feature into the subset of features according to the *χ*^2^ test score and once more, a performance check is carried out using the GNB model on the subset of features. The same process is repeated till the point where the constructed subset of features approaches the full set of features. Lastly, a selection of the subset is made as the optimal subset of features showing the greatest performance. The ideal (i.e., optimal) subset of features is given to GNB to generate the best results. The formulation of GNB model is as follows.

Naive Bayes (NB) is a set of supervised predictive models known for their simplicity and effectiveness. These models learn the probabilities of an object with certain features belonging to a particular class or group, i.e., it is a group of probabilistic predictive models. These models are given the name “naive” because they make use of the naive assumption of independence, i.e., the models make the assumption that the occurrence of a certain feature is independent of the occurrence of other features. An NB model is based on Bayes theorem or rule, i.e., it evaluates the probability that a given instance belongs to a certain class. Given an instance *X* and a class label *θ*, using Bayes theorem, we can express the conditional probability *P*(*θ*|*X*) as a product of simpler probabilities using the naive independence assumption:

(3) $$\begin{array}{c} P\left(\left.\theta \right|f_{1},\ldots ,f_{n}\right)=\frac{P\left(\theta \right)P\left(f_{1},\ldots ,f_{n}\left|\theta \right.\right)}{P\left(f_{1},\ldots ,f_{n}\right)}, \end{array}$$

where (*f*_1_, *f*_2_,…, *f*_*n*_) denotes the features of the feature vector *X*. According to the naive independence assumption, we have

(4) $$\begin{array}{c} P\left(f_{i}\left|\theta \right.,f_{1},\ldots ,f_{i-1},f_{i+1},\ldots ,f_{n}\right)=P\left(f_{i}\left|\theta \right.\right). \end{array}$$

For all *i*, (3) will get the form:

(5) $$\begin{array}{c} P\left(\left.\theta \right|f_{1},\ldots ,f_{n}\right)=\frac{P\left(\theta \right)\prod_{i=1}^{n}P\left(f_{i}\left|\theta \right.\right)}{P\left(f_{1},\ldots ,f_{n}\right)}. \end{array}$$

As for a given instance, *P*(*f*_1_,…, *f*_*n*_) is constant. Thus, we can use the following classification rule:

(6) $$\begin{array}{c} P\left(\left.\theta \right|f_{1},\ldots ,f_{n}\right)\propto P\left(\theta \right)\overset{n}{\underset{i=1}{\prod }}P\left(f_{i}\left|\theta \right.\right),\\ \hat{\theta }=\arg\underset{\theta }{\max}P\left(\theta \right)\overset{n}{\underset{i=1}{\prod }}P\left(f_{i}\left|\theta \right.\right). \end{array}$$

For the estimation of the parameters in the NB model, i.e., *P*(*θ*) and *P*(*f*_*i*_|*θ*), maximum a posteriori (MAP) estimation is commonly used. The main idea is the same for different Naive Bayes models. However, different Naive Bayes models use different assumptions regarding the distribution of *P*(*f*_*i*_|*θ*). In case of the GNB model, the likelihood of the features is assumed to be Gaussian.

(7) $$\begin{array}{c} P\left(f_{i}\left|\theta \right.\right)=\frac{1}{\sqrt{2\pi \sigma_{\theta }}}\exp\left(\frac{-{\left(f_{i}-\mu_{\theta }\right)}^{2}}{2\sigma_{\theta }^{2}}\right), \end{array}$$

where the parameters *σ*_*θ*_ and *μ*_*θ*_ are estimated using maximum likelihood. In this paper, the performance of GNB is estimated on the HF disease dataset. To further enhance the performance of GNB, *χ*^2^ model is hybridized with it.

## 3. Evaluation Metrics and Validation Methods

### 3.1. Validation Methods

In this section, a binary classification problem is considered with two classes of diagnosis, i.e., healthy and patients who are prone to potential HF disease. Different studies have been conducted on the Cleveland heart disease dataset, and methods that achieved accuracies between 50% and 92.2% are reported in the literature. Most of these studies like [2, 23, 24] made use of a validation known as holdout, with a split of 70–30, i.e., training the proposed model with 70% dataset, and for the purpose of testing, 30% of the dataset is utilized. The methodology adopted in this paper for data portioning is the same as aforementioned.

### 3.2. Evaluation Metrics

The robustness of the proposed model is evaluated in this paper using different evaluation metrics including accuracy, specificity, sensitivity, and Matthews coefficient of correlation (MCC). The percentage of subjects that are classified correctly represents accuracy in the training or testing dataset, sensitivity on the other hand, and carries information about patients that are classified correctly, whereas the correctly classified healthy subjects denote specificity.

(8) $$\begin{array}{c} \text{Accuracy}=\frac{\text{TP}+\text{TN}}{\text{TP}+\text{TN}+\text{FP}+\text{FN}}. \end{array}$$

Here, the count of true positives is expressed as TP, the count of false positives is expressed as FP, the count of true negatives is expressed as TN, and the count of false negatives expressed as FN:

(9) $$\begin{array}{c} \text{sensitivity}=\frac{\text{TP}}{\text{TP}+\text{FN}},\\ \text{specificity}=\frac{\text{TN}}{\text{TN}+\text{FP}},\\ \text{MCC}=\frac{\text{TP}\times \text{TN}-\text{FP}\times \text{FN}}{\sqrt{\left(\text{TP}+\text{FP}\right)\left(\text{TP}+\text{FN}\right)\left(\text{TN}+\text{FP}\right)\left(\text{TN}+\text{FN}\right)}}. \end{array}$$

MCC is the measure of quality of binary classification in machine learning. It can assume any value between the range of −1 and 1, where −1 shows the disagreement in total between prediction and observation, 1 shows a prediction that is perfect, and 0 refers to classification not more than a random prediction.

## 4. Experimental Results and Discussion

### 4.1. Experiment No. 1: Performance of the Proposed *χ*^2^-GNB Model

In this section, the experimental results of the proposed *χ*^2^-GNB are reported and discussed. After feature ranking by *χ*^2^ test, we obtained different subset of features. These subsets of features are applied sequentially to GNB predictive model for classification. The best accuracy of 93.33% is achieved on testing data with subset of features having *n*=9, i.e., 9 features. As can be seen in Table 3, a training accuracy of 84.05% is achieved for the (optimal) subset of features, while sensitivity and specificity are reported to be 87.80% and 97.95%, respectively. From the perspective of machine learning, the ideal (optimal) subset of features apart from enhancing performance of the GNB model also decreases the complexity of the GNB model. This thereby, leads to a reduction in the GNB models training time. Moreover, the subset of features with *n*=9 includes *f*_2_, *f*_3_, *f*_7_, *f*_8_, *f*_9_, *f*_10_, *f*_11_, *f*_12_, and *f*_13_. The classification results achieved for different subsets of features are reported in Table 3. The last row in the table represents the case when no features are selected, i.e., all the features are applied to the GNB model, and the prediction accuracy of 90% is achieved. Hence, it proves that the developed *χ*^2^-GNB model enhances the potential of GNB model by 3.3%.

**Figure 1:**
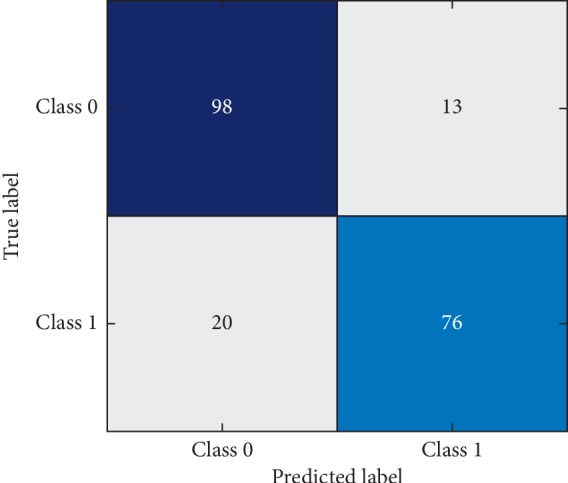
Confusion matrix of training data.

**Figure 2:**
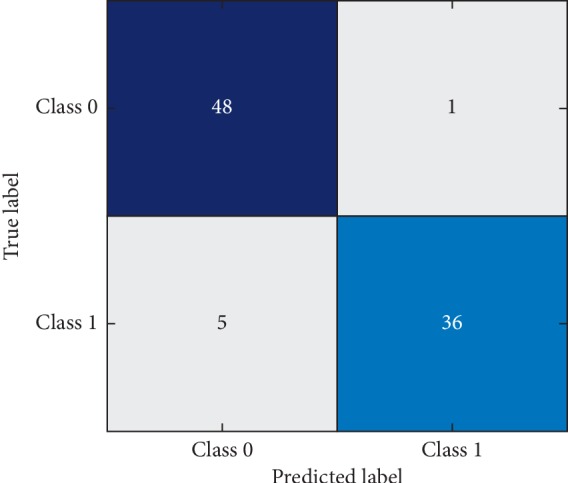
Confusion matrix of testing data.

To get information about the statistics of correctly classified healthy subjects, patients and misclassification rate and confusion metrics are drawn in Figures 1 and 2 for training data and testing data, respectively. The horizontal class labels denote the predicted labels, while the vertical labels represent true labels. The testing data contain 90 samples out of which 41 are patients and 49 are healthy subjects. From the confusion matrix, the proposed model can correctly detect 36 patients, thus yielding sensitivity of 87.80% which is in accordance with sensitivity reported in Table 3. Similarly, out of 49 healthy subjects, the proposed method is capable of correctly classifying 48 subjects. Hence, the proposed method yields specificity of 97.95%.

The training data consist of 207 samples out of which 96 are patients and remaining are healthy subjects. The proposed model correctly classifies 76 patients, while 20 patients are misclassified. On the other hand, 98 healthy subjects are correctly classified, while 13 are misclassified.

### 4.2. Experiment No. 2: Comparative Study of the Proposed *χ*^2^-GNB Model with Other State-of-the-Art Ensemble Models and Support Vector Machines

To prove how effective the *χ*^2^-GNB method is, we perform a study by comparing it with other existing models of machine learning. These existing models encompass the support vector machine (SVM) with RBF and linear kernel and the ensemble models. The extra tree, often referred to as the randomized decision tree, Adaboost, and the random forest (RF) are the ensemble models that are considered for the comparison purposes. With the use of grid search algorithm, these models are therefore searched for the ideal (optimal) values of their hyperparameters.  Table 4 presents the performance of each optimized model.

In Table 4, the number of trees in the forest is denoted by *N*_*e*_, a hyperparameter for the RF model. In case of Adaboost, the highest number of estimators for which termination occurs in boosting is denoted by the hyperparameter *N*_*e*_. Extra tree as an ensemble model makes use of averaging to enhance the accuracy of prediction and uses the number of randomized decision trees as a hyperparameter. With regards to extra tree, the number of trees used is represented by the hyperparameter *N*_*e*_. In terms of SVM, constant of the soft margin is denoted by *C*, and Gaussian Kernel width is denoted by *G*. Lastly, in the table, the size of the ideal (optimal) subset of features selected by the *χ*^2^ statistical model, in case of the *χ*^2^-GNB, is represented by *n*=9. It is clearly proved in the table that, with respect to performance, the proposed approach outperforms the different existing models (i.e., the SVM model with linear and RBF kernel and the ensemble models).

**Figure 3:**
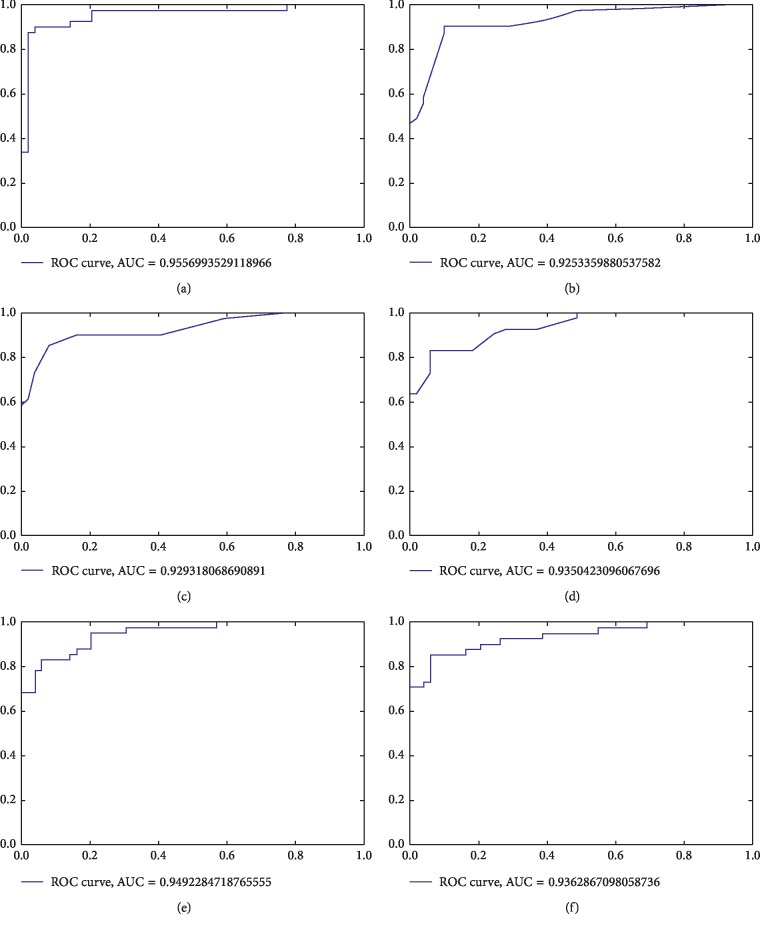
ROC charts of the *χ*^2^-GNB model and optimized SVM and ensemble models. ROC chart of the (a) proposed model, (b) Adaboost ensemble model, (c) extra tree ensemble model, (d) random forest, (e) linear SVM model, and (f) SVM (RBF) model.

Further, two more metrics, i.e., receiver operating characteristic (ROC) curve and area under the curve (AUC) are used to conduct more investigations on the effectiveness of the *χ*^2^-GNB model. For different thresholds, the true positive rate (TPR) versus the false positive rate (FPR) is displayed graphically by the ROC chart. This indicates that an ideal ROC curve of the plot is one that resides at the top-left corner of the graph. More precisely, we can state that the larger the area under the curve of the ROC chart, the better the developed model [25].  Figure 3 displays the ROC chart of the *χ*^2^-GNB model and other models of machine learning. From the chart in Figure 3(a), the *χ*^2^-GNB model indicates a 0.955 area under the curve (AUC). An optimized Adaboost model has a 0.925 AUC as depicted in Figure 3(b), whereas a 0.929 AUC as shown in Figure 3(c) is indicated by the optimized extra tree model. Furthermore, from Figure 3(d), the random forest model (optimized) indicates an AUC of 0.935. With the support vector machine (SVM), the linear kernel indicates a 0.949 AUC, whereas the RBF kernel indicates an AUC of 0.936 as displayed, respectively, in Figures 3(e) and 3(f). It is clearly proven as shown in the ROC charts that the *χ*^2^-GNB approach outperforms the other existing optimized ensemble and SVM approaches.

### 4.3. Comparison of the Proposed Method with Other Well-Known Machine Learning Methods Proposed Earlier

In this section, we compare our results in terms of HF disease detection accuracy with the results of previously proposed methods in literature.  Table 5 briefly discusses other methods developed for HF risk prediction and compares their accuracies with the model developed in this study. It can be seen that the newly developed model shows better HF risk prediction accuracy than many of the previous methods.

## 5. Conclusion

In this paper, on the basis of *χ*^2^ statistical model and GNB, we proposed a feature-driven decision support system for HF disease prediction. It was shown that the newly developed model, i.e.,*χ*^2^-GNB, enhanced the performance of the conventional GNB model. In order to evaluate the robustness of the *χ*^2^-GNB model, six evaluation metrics were used, i.e., sensitivity, accuracy, specificity, ROC, AUC, and MCC. It was observed that the proposed model improved the performance of GNB model by 3.33%. Moreover, a comparative analysis based on prediction accuracy between the *χ*^2^-GNB model and other previously reported methods was carried out. It was shown through experimental results that the *χ*^2^-GNB model outperformed the state-of-the-art ensemble models, support vector machines and many previously proposed methods.

## Figures and Tables

**Table 1:** Commonly used HF features of the dataset.

Feature no.	Feature description	Feature code
1	Age (AGE)	*f*_1_
2	Sex (SEX)	*f*_2_
3	Chest pain type (CPT)	*f*_3_
4	Resting blood pressure (RBP)	*f*_4_
5	Serum cholesterol (SCH)	*f*_5_
6	Fasting blood Sugar (FBS)	*f*_6_
7	Resting electrocardiographic results (RES)	*f*_7_
8	Maximum heart rate achieved (MHR)	*f*_8_
9	Exercise induced angina (EIA)	*f*_9_
10	Old peak (OPK)	*f*_10_
11	Peak exercise slope (PES)	*f*_11_
12	Number of major vessels colored by fluoroscopy (VCA)	*f*_12_
13	Thallium scan (THA)	*f*_13_

**Table 2:** Table to compute *χ*^2^ test score.

	Positive class	Negative class	Total
Feature *f*_*i*_ occurs	*α*	*β*	*α*+*β*=*μ*
Feature *f*_*i*_ does not occur	*λ*	*γ*	*λ*+*γ*=*τ* − *μ*
*Total*	*α*+*β*=*ω*	*β*+*γ*=*τ* − *ω*	*τ*

The sum of instances comprising feature *f*_*i*_ is denoted by *μ*, the sum of instances without feature *f*_*i*_ is denoted by *τ* − *μ*, the sum of instances that are positive is expressed as *ω*, and the sum of instances that are negative are represented by *τ* − *ω*. Let the observed values be *α*, *β*, *λ*, and *γ* with the expected values *E*_*α*_, *E*_*β*_, *E*_*λ*_, and *E*_*γ*_. Based on the hypothesis that the two events are independent, the expected value can be evaluated as follows:

**Table 3:** Results of different subsets of features for the heart disease dataset.

n	*Acc*_test_	*Acc*_train_(%)	Spec. (%)	Sens. (%)	MCC
1	78.88	75.36	81.63	75.60	0.573
2	88.11	78.26	79.59	82.92	0.622
3	84.44	81.64	89.79	78.04	0.686
4	86.66	79.71	91.83	80.48	0.732
5	86.66	80.19	93.87	78.04	0.734
6	86.66	80.67	89.79	82.92	0.730
7	90.00	82.12	91.83	87.80	0.798
8	90.00	83.57	93.87	85.36	0.799
**9**	**93.33**	**84.05**	**97.95**	**87.80**	**0.868**
10	90.00	81.64	93.87	85.36	0.799
11	90.00	82.60	93.87	85.36	0.599
12	90.00	84.05	93.87	85.36	0.799
13	90.00	82.12	93.87	85.36	0.799

**Table 4:** Experimental results of other optimized machine learning models.

Model	Hyperparameters	*Acc*_test_	Spec.	Sens.	MCC
SVM (linear)	*C*=0.055	90	93.87	85.36	0.799
SVM (RBF)	*C*=5, *G*=0.2	90	93.87	85.36	0.799
Adaboost	*N*_*e*_=4	88	89.79	87.80	0.776
Extra tree	*N*_*e*_=11	88	89.79	87.80	0.776
Random forest	*N*_*e*_=50	88	93.87	82.92	0.777
*Proposed*	*n*=9	**93.33**	**97.95**	**87.80**	**0.868**

**Table 5:** Details of other machine learning methods proposed for HF prediction and their obtained HF prediction accuracies.

Study (year)	Method	Accuracy (%)
ToolDiag, RA [26]	IB1-4	50.00
WEKA, RA [26]	InductH	58.50
ToolDiag, RA [26]	RBF	60.00
WEKA, RA [26]	FOIL	64.00
ToolDiag, RA [26]	MLP + BP	65.60
WEKA, RA [26]	T2	68.10
WEKA, RA [26]	1R	71.40
WEKA, RA [26]	IB1c	74.00
WEKA, RA [26]	K	76.70
Robert Detrano [26]	Logistic regression	77.00
Cheung (2001) [27]	C4.5	81.11
Cheung (2001) [27]	Naive Bayes	81.48
Cheung (2001) [27]	BNND	81.11
Cheung (2001) [27]	BNNF	80.96
WEKA, RA [26]	Naive Bayes	83.60
Ster and Dobnikar [28]	Fisher discriminant analysis	84.2
Ster and Dobnikar [28]	Linear discriminant analysis	84.5
Ster and Dobnikar [28]	Naive Bayes	82.5–83.4
Polat et al. (2005) [18]	AIRS	84.50
Ozsen et al. (2005) [29]	Kernel functions with AIS	85.93
Kahramanli and Allahverdi (2008) [30]	Hybrid neural network system	86.8
Polat et al. (2006) [1]	Fuzzy-AIRS-Knn-based system	87.00
Özşen and Güneş (2009) [19]	Modified artificial immune system	87.43
Das et al. (2009) [2]	Neural network ensembles	89.01
Jankowski and Kadirkamanathan (1997) [31]	IncNet	90.00
Kumar (2011) [32]	ANFIS	91.18
Samuel et al. (2017) [20]	ANN-fuzzy-AHP	91.10
Kumar (2012) [33]	Fuzzy resolution mechanism	91.83
Ali et al. (2019) [21]	Stacked and optimized SVMs	92.22
Paul et al. (2018) [23]	Adaptive weighted fuzzy system ensemble	92.31
*Proposed method (2018)*	*χ* ^2^-GNB	**93.33**

## Data Availability

The data used in the paper are publicly available at the UCI machine learning repository.
